# Structure of bacterial oligosaccharyltransferase PglB bound to a reactive LLO and an inhibitory peptide

**DOI:** 10.1038/s41598-018-34534-0

**Published:** 2018-11-02

**Authors:** Maja Napiórkowska, Jérémy Boilevin, Tamis Darbre, Jean-Louis Reymond, Kaspar P. Locher

**Affiliations:** 1 Institute of Molecular Biology and Biophysics, ETH Zurich, Zurich, Switzerland; 2 Department of Chemistry and Biochemistry, University of Bern, Bern, Switzerland

## Abstract

Oligosaccharyltransferase (OST) is a key enzyme of the *N*-glycosylation pathway, where it catalyzes the transfer of a glycan from a lipid-linked oligosaccharide (LLO) to an acceptor asparagine within the conserved sequon N-X-T/S. A previous structure of a ternary complex of bacterial single subunit OST, PglB, bound to a non-hydrolyzable LLO analog and a wild type acceptor peptide showed how both substrates bind and how an external loop (EL5) of the enzyme provided specific substrate-binding contacts. However, there was a relatively large separation of the substrates at the active site. Here we present the X-ray structure of PglB bound to a reactive LLO analog and an inhibitory peptide, revealing previously unobserved interactions in the active site. We found that the atoms forming the *N*-glycosidic bond (C-1 of the GlcNAc moiety of LLO and the –NH_2_ group of the peptide) are closer than in the previous structure, suggesting that we have captured a conformation closer to the transition state of the reaction. We find that the distance between the divalent metal ion and the glycosidic oxygen of LLO is now 4 Å, suggesting that the metal stabilizes the leaving group of the nucleophilic substitution reaction. Further, the carboxylate group of a conserved aspartate of PglB mediates an interaction network between the reducing-end sugar of the LLO, the asparagine side chain of the acceptor peptide, and a bound divalent metal ion. The interactions identified in this novel state are likely to be relevant in the catalytic mechanisms of all OSTs.

## Introduction

*N*-protein glycosylation is a post-translational modification that is conserved in all domains of life^1–7^. The addition of a glycan to a protein can profoundly affect its structure, function, and targeting. In eukaryotes *N*-glycosylation can be important in protein trafficking, cellular signalling or self-non-self interactions^1,8,9^. In bacteria, including the food pathogen *Campylobacter jejuni*, *N-*glycans improve host cell- adhesion and colonization, indicating their role in virulence^5,10^.

Although bacteria and eukaryotes display different glycan structures attached to asparagine residues, their *N*-glycosylation pathways are thought to be homologous and follow a conserved mechanism^11^. The oligosaccharide is first assembled on a pyrophosphate-lipid carrier and then transferred *en bloc* to conserved sequons (N-X-S/T) of secretory proteins. The reaction is catalyzed by an integral-membrane oligosaccharyltransferase (OST). Whereas the OST of higher eukaryotes is a multiprotein complex with STT3 being the catalytic subunit, archaea, kinetoplastids and bacteria have single-subunit OST (ssOST) enzymes that are homologous to STT3^4,12–14^. Although OSTs have been found to show some relaxed specificity with respect to the oligosaccharide donor, bacterial and archaeal enzymes can transfer a much vaster array of glycans compared to their eukaryotic counterparts^3,12,15–18^.

X-ray structures of ssOSTs of *Campylobacter lari* (PglB) and *Archaeoglobus fulgidus* (AglB) have revealed the fold of the enzyme^13,14,19,20^. Extensive *in vitro* studies and a high resolution structure of PglB bound to a wild type peptide and a non-hydrolyzable LLO provided many mechanistic details of peptide and LLO recognition^15,16,21–23^. In particular, it was found that the external loop 5 (EL5), present in all OST enzymes, provided crucial interactions to bound substrates: Whereas the N-terminal half primarily contacted bound LLO, the C-terminal half provided key contacts to bound acceptor peptide. Engagement and disengagement of EL5 appeared essential for substrate binding and product release^19,23^.

Despite the available structural data, the exact mechanism of glycan transfer is not fully understood. For example, details of how the substrates interact and how the amido group of the acceptor asparagine is activated, are lacking. Specifically, the previously solved ternary complex structure revealed an arrangement of bound substrates that was evidently not very close to the transition state given that the distance between the atoms that would form the glycosidic bond was ~6 Å. Here we present the X-ray structure of PglB in complex with an inhibitory peptide and a synthetic reactive LLO analog. The structure captures a new intermediate of the glycosylation reaction where the substrates are significantly closer, identifying new contacts with conserved residues in the catalytic site. The new structural evidence reveals previously unknown interactions between PglB, LLO, acceptor peptide and the bound divalent metal ion.

## Results

### Strategy to trap a distinct ternary complex

**Figure 1:**
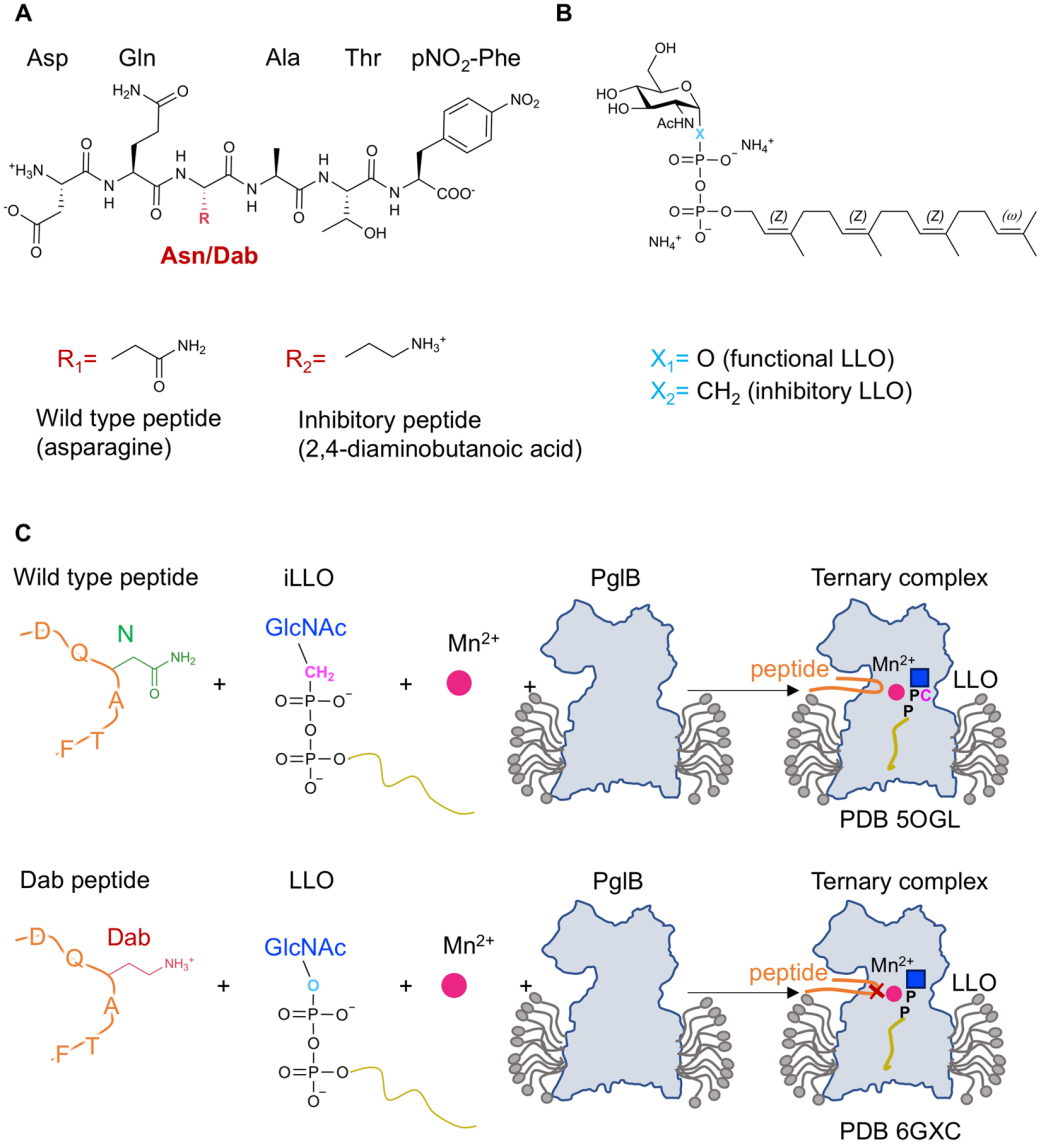
Strategies to trap distinct intermediate states of the PglB-catalyzed glycosylation reaction. (**A**) Structure of the substrate and inhibitory peptides used in crystallization experiments. The red ‘R’ denotes a side chain of either asparagine or 2,4-diaminobutanoic acid (Dab). (**B**) Structure of the synthetic LLO analogs used in crystallization experiments. The blue ‘X’ denotes the linkage between C-1 of the GlcNAc moiety and the phosphorous atom of the first phosphate group. (**C**) Schematic of two strategies pursued to trap ternary complexes of PglB using different peptide and LLO analogs. Top: method used previously to co-crystallize PglB with a wild type peptide and a non-hydrolyzable LLO analog (iLLO)^19^. Bottom: method used in this study to co-crystallize PglB with an inhibitory, Dab-containing peptide and a reactive LLO analog. The outline of *C*. *lari* PglB in a detergent micelle is shown in grey, peptide in orange and the LLO analogs are represented by a blue square for the *N*-acetyl-glucosamine (GlcNAc) moiety, a black ‘P’ for the phosphate group and a gold line for the lipid tail. The non-hydrolyzable LLO analog and the inhibitory peptide are marked with a pink ‘C’ and a red ‘X’, respectively.

**Figure 2:**
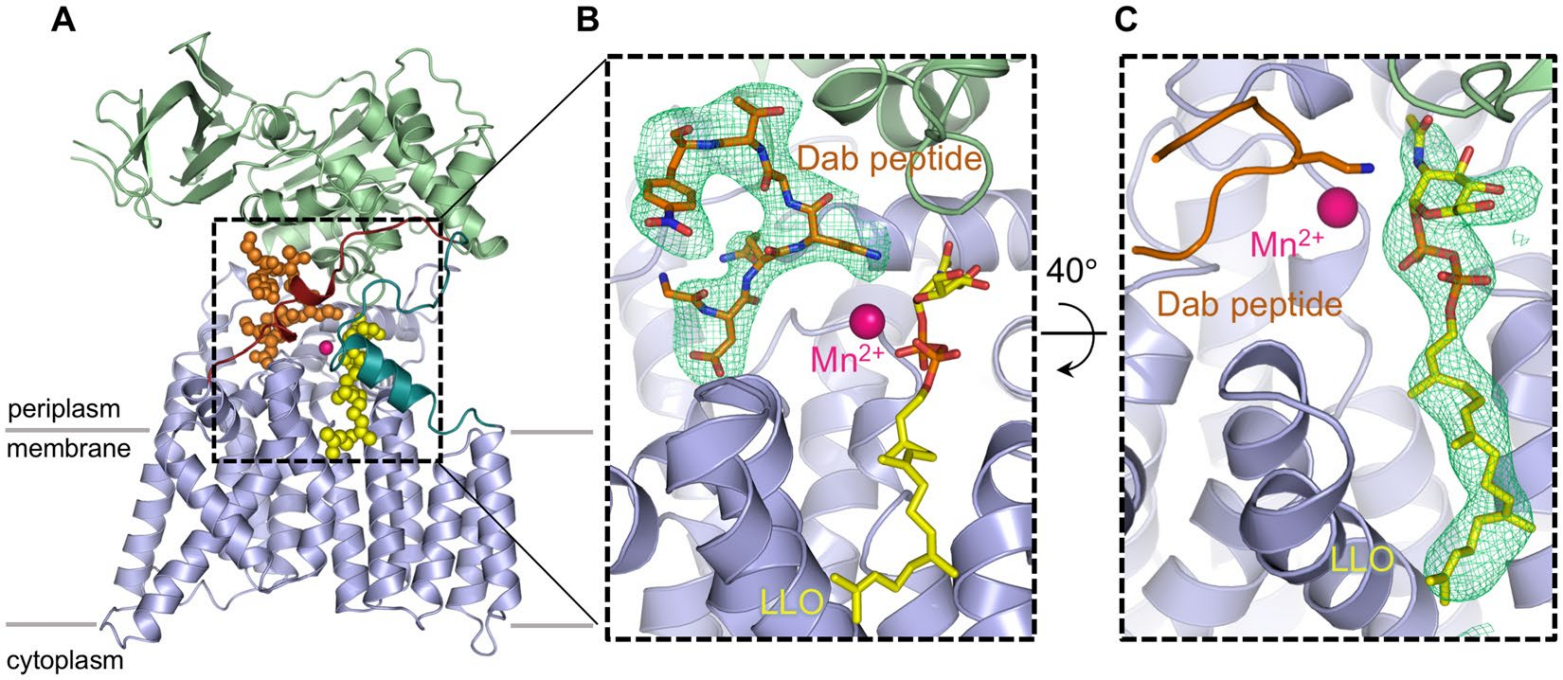
Structure of the ternary complex with inhibitory Dab peptide and reactive LLO. (**A**) cartoon representation of PglB with the transmembrane domain colored in light blue, periplasmic domain in light green, and N-terminal and C-terminal part of EL5 in turquoise and dark red, respectively. The LLO and Dab peptide are shown as yellow and orange spheres, respectively. (**B**) Close-up view of the PglB active site. EL5 was removed for clarity. The LLO and Dab peptide are shown as yellow and orange sticks and the divalent metal ion in pink. A polder omit map contoured at 4.0 $\sigma$ around bound Dab peptide is shown as green mesh. (**C**) close-up view of the PglB active site rotated 40 ° relative to that in B. A polder omit map contoured at 4.0 $\sigma$ around bound LLO is shown as green mesh. The Dab peptide is shown as ribbon.

Two strategies can in principle be pursued to trap ternary complex states of OST (Fig. 1). The first, which was the basis of the previously published structure^19^, was to co-crystallize PglB with wild type peptide (DQNAT(pNO_2_-F)) and a non-hydrolyzable, phosphonate-containing LLO analog (ωZZZ-PPCH_2_-GlcNAc). DQNATF is an optimal acceptor sequon for *C*. *jejuni* PglB and the phosphonate-containing LLO analog was identified as a competitive inhibitor (Fig. 1)^14,19,24^. In the resulting ternary complex structure we found that the phosphonopyrophosphate group was not coordinated by the catalytic Mn^2+^ ion and that the substrates were distantly located (~6 Å apart). We hypothesized that this gap was due to the use of a phosphonopyrophosphate rather than a pyrophosphate group and that using a reactive LLO might reveal a bound state closer to the transition state of the reaction. We therefore pursued a second strategy to form a ternary complex by incubating PglB with a functionally competent, synthetic LLO analog and an inhibitory peptide (Fig. 1). We chose 2,4-diaminobutanoic acid (Dab) to replace the acceptor asparagine, because Dab-containing peptides were previously shown to act as competitive inhibitors of bacterial and eukaryotic OSTs at physiological pH (Fig. 1A)^21,25,26^. We investigated whether the Dab-containing peptide and the functional LLOs showed activity with PglB at pH values used to crystallize it (pH ~8.9) and did not detect any glycosylation of the Dab-containing peptide, demonstrating that no glycan transfer occurs even after very long incubations. We therefore used this Dab-containing peptide for crystallization experiments. We used the reactive synthetic LLO analog ωZZZ-PP-GlcNAc, which is chemically very similar to the wild type LLO except that it contains a GlcNAc rather than di-*N*-acetyl-bacillosamine as a reducing-end sugar (Fig. 1)^19^. This LLO compound exhibited the highest turnover rate of all of the water-soluble synthetic LLO analogs tested^19,27^. The resulting ternary complex was crystallized and the structure determined at 3.4 Å resolution (Fig. 2 and Table 1).

**Table 1:** Data collection and refinement statistics (molecular replacement).

	Ternary complex^a^
**Data collection**	
Space group	P2_1_2_1_2_1_
Cell dimensions	
*a*, *b*, *c* (Å)	83.81, 116.54, 173.89
α, β, γ (°)	90.0, 90.0, 90.0
Resolution (Å)	40.74–3.40 (3.49–3.40)*
R_meas_	0.069 (1.44)*
I/*σ*	23.25 (1.95)*
CC_1/2_	0.999 (0.676)*
Completeness (%)	92.1 (36.7)*
Redundancy	12.4 (4.8)*
**Refinement**	
Resolution (Å)	40.74–3.40
No. reflections	24002 (2377)*
R_work_/R_free_	0.2492/0.2831
No. non hydrogen atoms	5931
Protein	5866
Ligands	65
B-factors (Å^2^)	
Protein	110.17
Ligands	103.73
R.m.s. deviations	
Bond lengths (Å)	0.008
Bond angles (°)	1.13

Values shown correspond to statistics after anisotropy correction.

^a^Data collected from one crystal.

*Values in parentheses are for highest-resolution shell.

### LLO binding and the role of Mn^2+^

**Figure 3:**
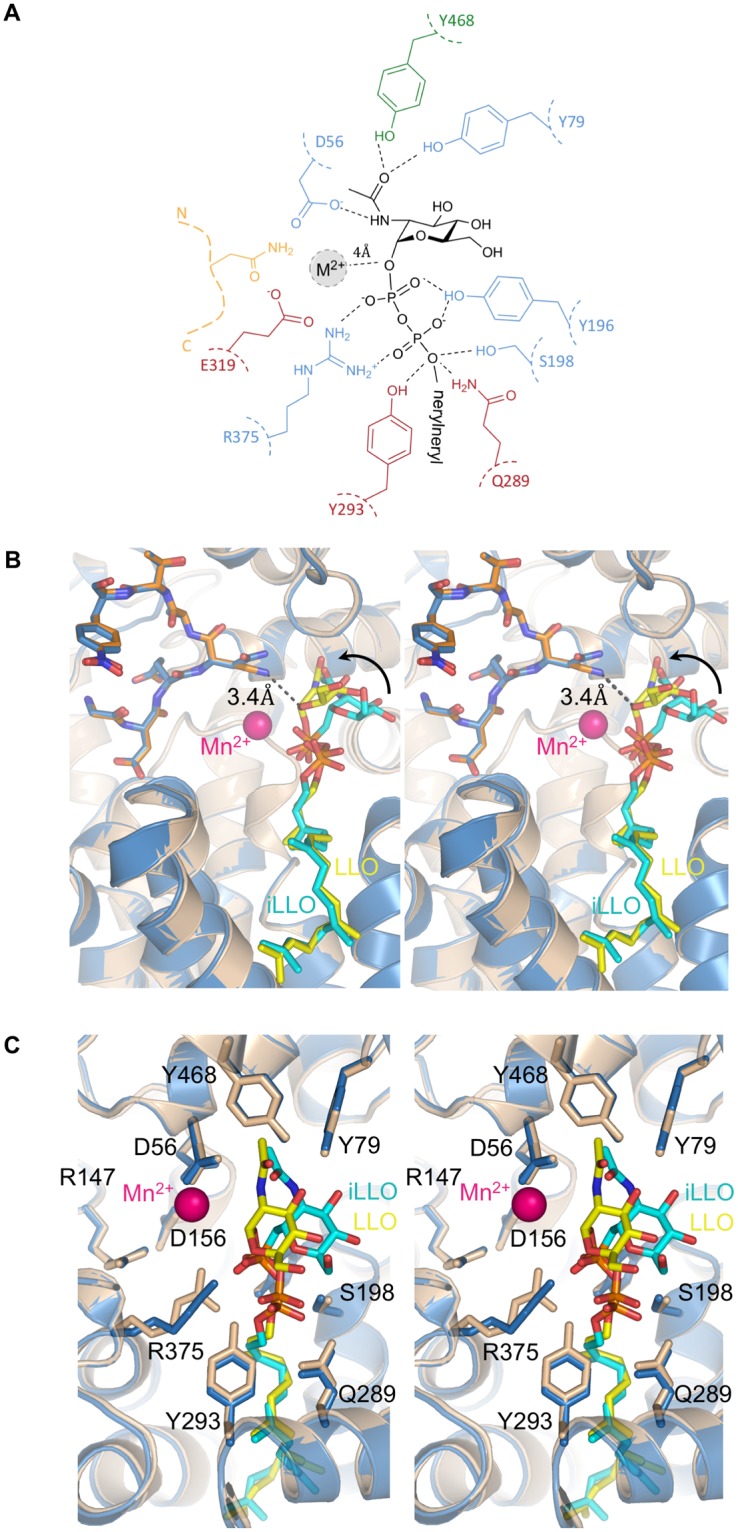
Interactions between PglB and bound LLO (**A**) Schematic of the interactions. Periplasmic, transmembrane and EL5 residues are shown in green, blue and red, respectively, and labeled. The acceptor peptide is shown in orange. The conserved R375 and Y196 residues interact with both phosphate groups. A previously unobserved interaction between the side chain of D56 and the *N*-acetyl group of the GlcNAc moiety is suggested by the structure. (**B**) Stereo view of the superposition of both ternary complexes. The new ternary complex PglB–LLO–Dab-peptide is shown in light brown with the reactive LLO and inhibitory Dab peptide shown in orange and yellow sticks, respectively. The previously reported ternary complex (PDB 5OGL) is shown in blue with the non-hydrolyzable LLO analog (iLLO) and WT acceptor peptide shown in cyan and dark blue sticks, respectively. The divalent metal ion is shown as a pink sphere. The black dashed line represents the distance between the reactive LLO and the amino group of the Dab residue. (**C**) Stereo view of the LLO binding site rotated 90° relative to that of B. The conserved residues in the catalytic site are shown as sticks. The GlcNAc moiety is positioned closer to the Mn^2+^ ion and residue D56.

Whereas the overall structure of PglB bound to the reactive LLO and inhibitory peptide is very similar to the previously determined ternary complex, there are important differences (Fig. 2, Supplementary Fig. 1)^19^. The key difference is that the two substrates are closer in the new ternary complex, with a distance of ~3.4 Å between carbon C-1 of the GlcNAc moiety of reactive LLO and the –NH_2_ group of the Dab residue, representative of the atoms that would form an *N*-glycosidic bond if Dab was replaced by Asn. In contrast, the equivalent distance between the GlcNAc of non-reactive LLO and the acceptor asparagine was ~6 Å in the previously determined structure^19^. The closing of the distance is mainly due to a shift of the reducing-end GlcNAc and is probably due to new contacts formed. Whereas the lipid tail of the LLO analog has an indistinguishable conformation to the previous ternary complex, the pyrophosphate and GlcNAc moieties have shifted and interact differently with the surface of PglB in the new structure (Fig. 3). We now observe continuous electron density between the pyrophosphate group of LLO and the catalytic Mn^2+^, with the shortest distance ($\sim 4$  Å) observed between Mn^2+^ and the oxygen linking C1 of GlcNAc and the pyrophosphate moiety (Fig. 3A, Supplementary Fig. 2). This indicates that the divalent metal ion, instead of being coordinated by water molecules, interacts with the pyrophosphate group. The structure also revealed different interactions between the pyrophosphate of the LLO and two essential catalytic residues, R375 and Y196. They appear to form hydrogen bonds with both phosphate groups, not just with the phosphate directly attached to the GlcNAc, as was observed for the ternary complex with non-reactive LLO (Fig. 3). This may explain why mutation of either of these residues to alanine abolished the glycosylation activity of PglB, but not binding of acceptor peptide^19,22^. Our structure also revealed contacts between the reducing-end sugar and PglB that were not observed before. In the previous ternary complex, the *N*-acetyl group of the reducing-end GlcNAc was at a distance of ~3.1 Å from Y468 but was not close enough to interact with D56 (distance of 5.2 Å). In the present structure, the *N*-acetyl group can form hydrogen bonds with the side chains of both D56 and Y468. The carbonyl oxygen is placed at a distance of 2.2 Å from the hydroxyl oxygen of Y468, whereas the nitrogen is at a distance of 3.3 Å from the carboxyl group of D56. Both D56 and Y468 have previously been demonstrated to be essential for PglB activity, but D56 was only shown to be involved in Mn^2+^ coordination and peptide binding^14,19^. Because both D56 and Y468 are strictly conserved catalytic residues, the newly identified interactions are likely relevant for all OST enzymes.

### Mobility of the reducing-end GlcNAc in the catalytic site

**Figure 4:**
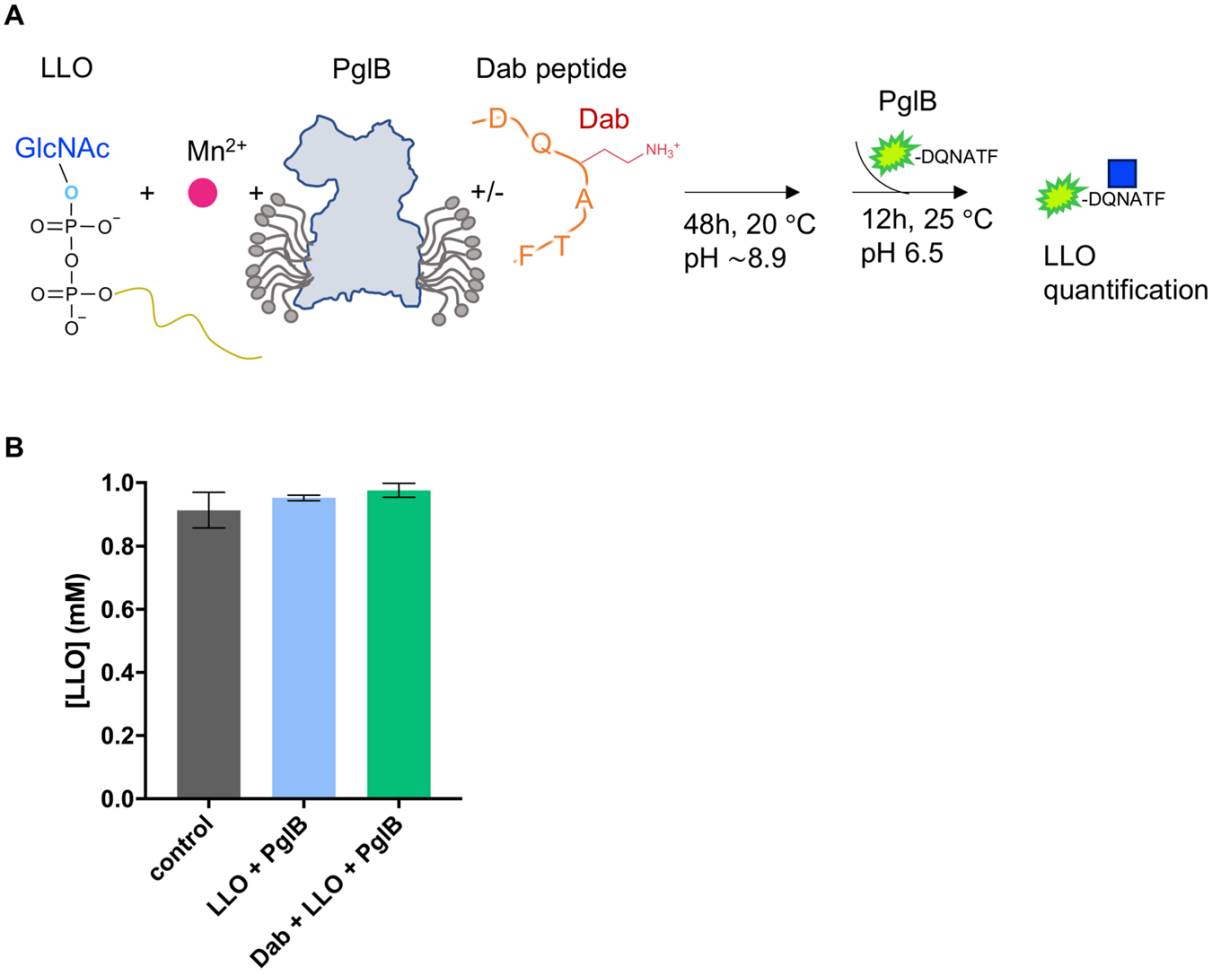
Stability of LLO at buffer conditions used for crystallization. (**A**) Schematic representation of the *in vitro* assay used to verify the stability of the reactive LLO in the crystallization conditions. LLO was incubated with PglB for 48 h at 20 °C in the presence or absence of Dab peptide and the amount of LLO was quantified. As a control, non-incubated LLO was used. (**B**) Quantification of the reactive LLO using an *in vitro* glycosylation assay. Data represents technical replicates and error bars indicate s.d. (n = 3).

The electron density for the lipid tail and the pyrophosphate group of reactive LLO was stronger than that for the GlcNAc moiety, unlike in the previous ternary complex with non-reactive LLO where the quality of the density for the LLO was similar throughout^19^. This suggests that either the sugar moiety of the reactive LLO molecule is more mobile, or that the LLO may have been hydrolyzed in the crystallization conditions, which would result in a mixture of LLO and lipid-pyrophosphate. Both PglB and yeast octameric OST were previously reported to exhibit hydrolytic activity and produce free oligosaccharides (fOS) in the absence of the acceptor sequon^28–30^. We therefore tested the stability of LLO by incubating it in crystallization conditions in the presence of PglB and the presence or absence of Dab peptide. We then quantified the amount of LLO left using an *in vitro* glycosylation assay, where the samples were diluted and incubated with freshly purified PglB and an excess of fluorescently labeled wild type peptide over LLO (Fig. 4A)^19,21,22^. We did not detect any loss of LLO in the crystallization conditions over a period of 48 hours, which suggests that the intact LLO analog, and not its hydrolyzed derivative, are present in the co-crystal (Fig. 4B). The discrepancy between our results from *in vitro* experiments and previous studies remains unresolved^28,29^. Because there is no LLO hydrolysis in our experiment, the slightly weaker density for GlcNAc could be explained by an increased mobility of the GlcNAc moiety of the reactive LLO. We speculate that for wild type heptasacccharide-containing LLO the presence of extra sugar moieties would form additional interactions with PglB, likely decreasing the mobility of the reducing-end sugar.

### Binding of the inhibitory peptide

The position of the backbone of the inhibitory peptide is unaltered relative to that of the wild type peptide in the previous ternary complex, validating the use of the Dab-containing peptide as a substrate mimic^19^. The Dab side chain reaches the catalytic site where it can form hydrogen bonds with three conserved and catalytically essential residues, the side chains of D56 and E319 and the main chain carbonyl oxygen of G482 (Fig. 3 and Supplementary Fig. 2)^14,21,22,31^. It was previously shown that replacing the acceptor asparagine with Dab resulted in a 10- fold decrease in peptide affinity^21^. This suggests that the presence of the carbonyl oxygen in the acceptor asparagine side chain, and the absence of a charge on the –NH_2_ group, produce favourable interactions with the enzyme that contribute to the binding affinity but also allow glycan transfer. However, when comparing the Dab-peptide- to the wild type-peptide-bound structure of PglB, there are no specific interactions (hydrogen bonds) between PglB and the carbonyl group of the acceptor asparagine that would contribute significantly to the increased peptide affinity, as only van der Waals interactions are observed (Fig. 3B)^19^. The exact chemical mechanism of increased binding and transfer activity, and the precise reason for why Dab is inhibiting, are therefore unknown.

## Discussion

The ternary complex presented here provides a first structural view into how OST interacts with a reactive LLO molecule. The captured intermediate is closer to the transition state of the glycosylation reaction than the previous ternary complex, because we observe an interaction between the pyrophosphate group of LLO and the Mn^2+^ ion, and the C-1 of GlcNAc is closer to the Dab side chain of the peptide than to the amide nitrogen in the previous structure^19^. The new structure also provides direct evidence that one of the roles of the divalent metal ion is the stabilization of the pyrophosphate, the leaving group of the substitution reaction. Similar roles were attributed to divalent metal ions in other glycosyltransferases (GTs) and were expected/predicted for OST^22,32–35^. Unexpectedly, however, the Mn^2+^ ion seems to be coordinated by the glycosidic oxygen atom rather than the negatively charged phosphate oxygens as observed in structures of GT-A superfamily members^32^. This suggests that the divalent metal ion might directly activate the glycosidic oxygen during the reaction by generating a reactive electrophile. This could compensate for the poor nucleophilicity of the carboxamide group of the acceptor asparagine and might even allow glycan transfer without amide activation. This hypothesis is in line with the observed inhibitory effect of Dab-containing peptides that cannot be glycosylated *in vitro*, which is probably due to the absence of a free electron pair in the protonated form. In contrast, it has been shown that a homoserine-containing peptide, which has the same number of methylene groups and therefore the same side chain length as Dab, is O-glycosylated^21^. The hydroxyl group of the homoserine analog, unlike the protonated Dab side chain, readily provides free electron pairs that can act as a nucleophile during the substitution reaction.

An *N*-acetyl group at the C-2 position of the reducing-end sugar is highly favoured by PglB and is strictly required for glycan transfer in eukaryotic OSTs^1,11,12,15,36,37^. Biochemical studies of yeast OST showed that minor modifications to this substituent, such as replacement of hydrogens with fluorines, reduced LLO binding and abolished glycosylation activity probably because the electron-withdrawing trifluoromethyl group made the neighbouring carbonyl oxygen a less efficient hydrogen bond acceptor^16^. The new ternary complex of PglB reveals hydrogen bonding interactions between the *N*-acetyl group of LLO and residues D56 and Y468 in the catalytic site of PglB. Because D56 also interacts with the acceptor peptide and the bound divalent metal ion, modification of the *N*-acetyl substituent is likely to affect the entire hydrogen bonding network, impairing glycan transfer^16^.

Although the new ternary complex reveals novel interactions, future functional and molecular dynamics studies are required to give insight into the exact role of the divalent metal ion in activation of the glycosidic oxygen of LLO. The recent cryo-EM structures of eukaryotic octameric OSTs revealed that the fold and key side chains of the catalytic STT3 subunit are conserved, demonstrating that PglB is an excellent model system to study the glycosylation reaction^19,37–39^. The structure presented here is therefore likely to prove valuable for future mechanistic interpretations not only of bacterial ssOSTs, but of OSTs from all domains of life.

## Materials and Methods

### Overexpression and purification of PglB

Overexpression and purification of PglB was performed as previously described^19^. Briefly, PglB was overexpressed in *Escherichia coli* BL21-Gold cells (DE3) (Strategene) at 37 °C in five-liter flasks using Terrific Broth medium supplemented with 1% glycerol (w/v). The cells were induced at 37 °C for 4 h at A600 of 3.0 by adding 0.1% arabinose (w/v). All following steps were carried out at 4 °C. Cells were harvested by centrifugation. Cells were resuspended in 25 mM Tris-HCl, pH 8.0, 250 mM NaCl and disrupted in a M-110-l microfluidizer (Microfluidics) at 15,000 p.s.i. chamber pressure. Membranes were pelleted by centrifugation at 100,000 g for 30 min and solubilized in 25 mM Tris-HCl, pH 8.0, 250 mM NaCl, 10% glycerol (v/v) and 1% N-dodecyl-β-D-maltopyranoside (w/v) (DDM, Anatrace) for 1.5 h. All subsequent purification buffers contained 0.016% DDM. PglB was purified on a nickel-nitrilotriacetic acid (Ni-NTA) Superflow affinity column (Qiagen) and desalted into 10 mM MES, pH 6.5, 100 mM NaCl, 3% glycerol (v/v), and 0.016% DDM. For crystallization experiments, desalted protein was concentrated to 6 mg mL^−1^ in an Amicon Ultra-15 concentrator (Milipore) with a molecular mass cutoff of 100 kDa and purified by size exclusion chromatography (Superdex 200 10/300, GE Healthcare) in desalting buffer. Collected fractions were pooled and concentrated to 12 mg mL^−1^.

### Protein crystallization

Ac-DQ(Dab)ATF(p-NO_2_)-NH2, nerylneryl-PP-GlcNAc and MnCl_2_ were added to the concentrated protein to the final concentration of 0.75 mM, 1.5 mM, 2 mM, respectively and incubated for 15 min at 4 °C. PglB was crystallized by vapor diffusion in hanging drops at 20 °C against a reservoir containing 100 mM glycine, pH 9.4, 50–200 mM magnesium acetate, and 27–31% PEG400 at the protein-to-reservoir ratio 2:1. Crystals usually appeared after 10–14 days and grew to full size within 3–4 weeks. Crystals were cryoprotected by stepwise addition of cryoprotectant PEG400 up to 30% final concentration and directly flash frozen in liquid nitrogen before data collection.

### Data collection and structural determination

The X-ray data was collected at the microfocus X06SA beamline at the Swiss Light Source (Villigen). The wavelength for data collection was 1.000 Å. All data were processed using XDS package^40^. Ellipsoidal truncation and anisotropic correction were applied to the data using the diffraction anisotropy server^41^. The ternary complex crystallized in the P2_1_2_1_2_1_ space group, containing one PglB molecule per asymmetric unit. The structure was solved by molecular replacement using PDB 5OGL as a model. The iterative model building and refinement was performed in Coot^42^ and Phenix^43^. Final refinement statistics and data collection are summarized in Table 1. Structural figures were drawn using PyMOL^44^.

### Quantification assay

LLO and PglB were incubated in the presence or absence of Dab peptide for 48 h at 20 °C. The mixture conditions replicated the crystallization conditions, where 10 mg mL^−1^ PglB, 1.5 mM LLO (nerylneryl-PP-GlcNAc), 0.75 mM inhibitory peptide (Ac-DQ(Dab)ATF{p-NO2}-NH2) in 10 mM MES pH 6.5, 3% glycerol, 100 mM NaCl, 0.016% DDM was added to 100 mM glycine pH 9.4, 28% PEG 400, 200 mM magnesium acetate in 2:1 ratio. The samples were diluted and the LLO was quantified against fluorescently labeled wild type peptide (DQNATF). The LLO dilutions were incubated for 12 h at 25 °C with 10 µM labeled peptide and purified protein in reaction buffer containing 10 mM MES pH 6.5, 100 mM NaCl, 3% glycerol 0.016% DDM, 10 mM MnCl_2_. The samples were analyzed by in-gel fluorescence and the ratio between glycosylated and non-glycosylated peptide was calculated using ImageJ. The concentration of LLO was determined in reactions where the wild type peptide was always present in excess over LLO. Data was fitted by linear regression in GraphPad PRISM 7.0.

### Synthesis of nerylneryl-PP-GlcNAc ((ωZZZ)-PP-GlcNAc))

The nerylneryl-PP-GlcNAc ((ωZZZ)-PP-GlcNAc) analog was synthesized according to previously reported procedures^19,27^. The synthetic strategy included three steps: the synthesis of GlcNAc α-phosphate, preparation of the nerylneryl-phosphate precursor, and finally the coupling of both monophosphates to the desired final pyrophosphate. Nerylneryl-PP-GlcNAc was shown to be pure by ^1^H, ^13^C, ^31^P NMR and ESI-HRMS.

## Electronic supplementary material

Supplementary Information

## Data Availability

Atomic coordinates were deposited in the Protein Data Bank under the accession code: PDB 6GXC.
