# Supplementary material for: Structure of bacterial oligosaccharyltransferase PglB bound to a reactive LLO and an inhibitory peptide
Source: Sci Rep. 2018 Nov 2;8:16297. doi: 10.1038/s41598-018-34534-0 (PMC6215017; doi:10.1038/s41598-018-34534-0)
Supplement: Supplementary file 1 — Supplementary Information [file 41598_2018_34534_MOESM1_ESM.pdf]

Supplementary Information

Structure of bacterial oligosaccharyltransferase PglB bound to a reactive LLO and an inhibitory peptide

Maja Napiórkowska[1], Jérémy Boilevin[2], Tamis Darbre[2], Jean-Louis Reymond[2] & Kaspar P. Locher[1*]

[1]Institute of Molecular Biology and Biophysics, ETH Zurich, Zurich, Switzerland.
[2]Department of Chemistry and Biochemistry, University of Bern, Bern, Switzerland
[*]Correspondence should be addressed to K.P.L. (locher@mol.biol.ethz.ch)

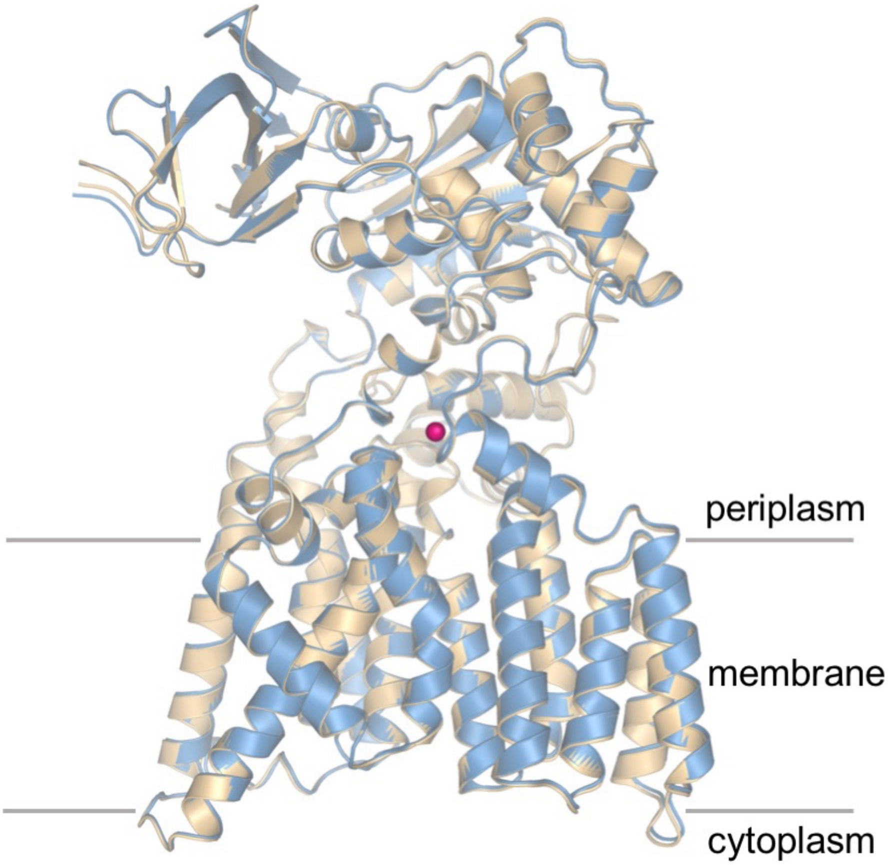

Supplementary Figure 1. Superposition of the structures of both PglB ternary complexes. The previously reported ternary complex (PDB 5OGL), shown in blue, aligned with the new ternary complex PglB–LLO–Dab-peptide, shown in light brown. The divalent metal ion is shown in pink. No significant conformational changes were observed.

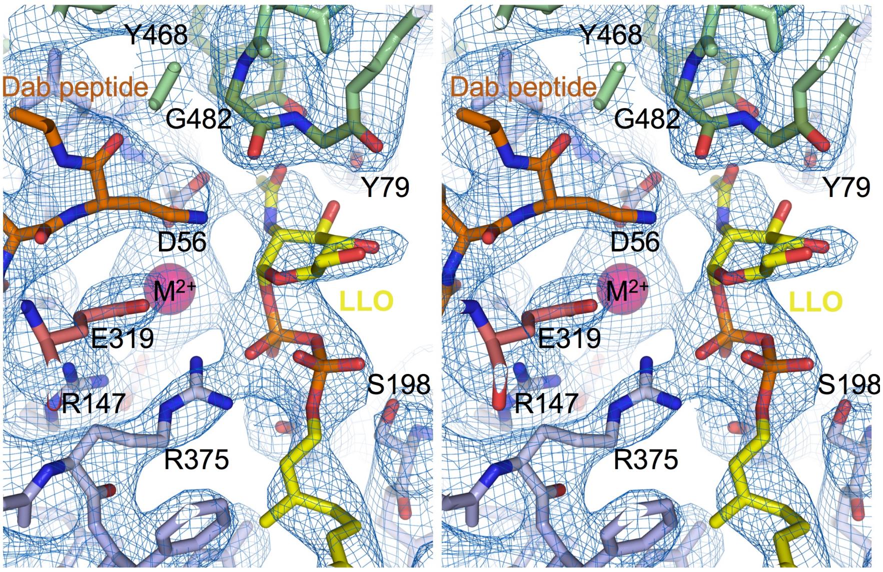

Supplementary Figure 2. Stereo view of the electron density map of the catalytic site. 2Fo-Fc electron density map is shown at 1.3 σ level. The LLO and Dab peptide are shown as yellow and orange sticks, the divalent metal ion is shown in pink. The periplasmic, transmembrane and residues in EL5 are colored in green, light blue and red, respectively. Unlike the previously described ternary complex structure (PDB 5OGL), continuous electron density was observed between the divalent metal ion and the pyrophosphate group.
